# Continuous removal of ethanol from dilute ethanol-water mixtures using hot microbubbles

**DOI:** 10.1016/j.cej.2021.130511

**Published:** 2021-11-15

**Authors:** Joseph Calverley, William B. Zimmerman, David J. Leak, H.C. Hemaka Bandulasena

**Affiliations:** a Department of Chemical Engineering, Loughborough University, LE11 3TU, United Kingdom; b Department of Chemical and Biological Engineering, University of Sheffield, S1 3JD, United Kingdom; c Department of Biology and Biochemistry, University of Bath, BA2 7AY, United Kingdom

**Keywords:** Microbubbles, Ethanol–water mixtures, Air stripping, Product inhibition, Bioethanol recovery

## Abstract

Product inhibition is a barrier to many fermentation processes, including bioethanol production, and is responsible for dilute product streams which are energy intensive to purify. The main purpose of this study was to investigate whether hot microbubble stripping could be used to remove ethanol continuously from dilute ethanol–water mixtures expected in a bioreactor and maintain ethanol concentrations below the inhibitory levels for the thermophile *Parageobacillus thermoglucosidasius* (TM242), that can utilize a range of sugars derived from lignocellulosic biomass. A custom-made microbubble stripping unit that produces clouds of hot microbubbles (~120 °C) by fluidic oscillation was used to remove ethanol from ~2% (v/v) ethanol–water mixtures maintained at 60 °C. Ethanol was continuously added to the unit to simulate microbial metabolism. The initial liquid height and the ethanol addition rate were varied from 10 to 50 mm and 2.1–21.2 g h^−1^ respectively. In all the experiments, ethanol concentration was maintained well below the inhibition threshold of the target organism (~2% [v/v]). This microbubble stripping unit has the potential to operate in conjunction with a 0.5–1.0 L fermenter to allow an ethanol productivity of 14.9–7.8 g L^−^^1^h^−1^ continuously.

## Highlights

• Continuous removal of ethanol from dilute mixtures using hot-microbubbles.
• Ethanol concentration maintained below the inhibition threshold for organism TM242.
• An ethanol productivity up to 14.9 g L^−1^h^−1^ was supported by the stripping unit.

## Nomenclature

Symbol: Description Unit
*A*: Integration parameter mol s^−1^
*B*: Integration parameter m^3^ s^−1^
*C*: Concentration % (v/v), g L^−1^ or moles L^−1^
*H*: Liquid height in vessel m
k: Constant representative of the system h^−1^
$M_{r}$: Molar mass g mol^−1^
*N*: Molar rate mol h^−1^
*P*: Pressure Pa
$\hat{P}$: Product generation rate of fermentation g L^−1^ h^−1^
$R_{feed}$: Dilution rate relative to the MSU liquid volume h^−1^
$R_{cir}$: Liquid circulation rate between the bioreactor and MSU L h^−1^
*R*: Universal gas constant J mol^−1^ K^−1^
*t*: Time h
*V*: Volume L
$\dot{V}$: Volumetric flow rate L h^−1^
*x*: Molar fraction % (mol/mol)

**Greek Symbols**
α: Volumetric evaporation rate L h^−1^
γ: Activity coefficient -
ρ: Density g L^−1^
χ: Constant representative of system-

**Subscripts**
*+*: Addition
*–*: Removal
*0*: Initial
∞: Steady-State
*Air*: Air
*E*: Ethanol
*F*: Feed
*L*: Liquid
*V*: Vapour/Condensate
*W*: Water

**Superscripts**
*: Equilibrium
0: Saturated/Standard conditions

## 1 Introduction

Bioethanol is a prime example of an alternative to petroleum fuels which has the potential to make the road transport sector more sustainable and environmentally friendly. However, the cost of production of bioethanol is still high compared to conventional petrol, due to the energy intensive processes involved and the cost of substrates used for production. Process intensification of biofuel production could make it more financially attractive. Generally, the production of bioethanol is done by microbial fermentation which is typically operated in batch [1], fed-batch [2] or continuous [3] mode. In batch systems concentrated sugar solutions are inoculated with a fermenting organism, typically the yeast *Saccharomyces cerevisiae,* which over time metabolises the sugars into a mixture of products, mainly carbon dioxide and ethanol under fermentative conditions. In fed-batch fermentation processes, yeast is inoculated into a moderately concentrated sugar solution, with a more concentrated sugar solution added over time. Fed-batch processes benefit from improved speed of fermentation as it removes substrate inhibition, where the high concentration of sugar reduces the metabolic rate over a certain threshold, typically ~150 g/L for *S. cerevisiae* [4]. In both cases, over the course of the fermentation the ethanol concentration reaches a point at which it begins to inhibit the growth of the organism until the fermentation process ceases completely [5]. Following the fermentation stage, the fermentation medium undergoes various unit operations such as distillation to recover and purify ethanol up to the azeotropic concentration and is then further processed up to fuel-grade ethanol using different separation techniques [6].

One of the main issues associated with current bioethanol production is the use of sugars derived from crop products that are also used as food. Use of farmland that can support food production, can result in a “food vs fuel” competition that leads to increases in food pricing, which is undesirable and unsustainable with the current population growth predictions [7], although this is still subject to debate [8]. For this reason, bioethanol that is produced from cellulosic biomaterials such as agricultural and food wastes has received significant attention. *Parageobacillus thermoglucosidasius* [9] has been identified as a promising microorganism that can metabolise complex sugars, including C5 sugars, available in pre-treated lignocellulosic feeds [10]. Genetic modification of the wild-type microorganism (producing strain TM242) has diverted fermentative metabolism from lactic acid primarily to ethanol production allowing fermentation of lignocellulose-derived feeds with high ethanol specificity and high rates that are comparable with the more commonly used microorganism *S. cerevisiae,* which cannot naturally metabolise C5 sugars. *P. thermoglucosidasius* is also thermophilic and has an optimum growth temperature of 60–65 °C [11]; therefore, it offers the additional advantage for *in situ* ethanol recovery due to the increased volatility of ethanol at high temperatures. However, this organism suffers from significant ethanol inhibition, with toxicity starting to exhibit above 2% (v/v).

Product inhibition associated with fermentations is a common problem, and although this study is focussed on ethanol removal, continuous removal of any volatile inhibitory product would lead to high productivity and lower separation and purification costs. One promising method for the removal of ethanol is gas stripping with direct contact evaporation (DCE). The previous works relating to direct contact evaporators are summarised by Ribeiro at al. [12]. Of particular note for their application to fermentation systems is that these operations are less prone to fouling as the contact area between phases is not permanent, they produce good quality mixing in the liquid phase, and they produce high quality heat and mass transfer. However, one obvious drawback with DCE is that these systems produce excessive foaming. This can be controlled by increasing the process pressure or by adding antifoaming agents [13], [14], [15]. Continuous removal of inhibitory products by DCE would permit fermentations to be carried out in fed-batch or continuous mode which would allow the use of highly concentrated substrate feeds. This would reduce the amount of water added [16], and hence the amount of dewatering required in the overall process. Thermophilic lignocellulosic fermentation of sugar cane bagasse with gas stripping has been reported by Kumar et al., but the ethanol productivity achieved was limited to 0.8–1.3 g L^−^^1^h^−1^ [11] which is low compared to the ethanol productivities of up to 3.2 g L^−^^1^h^−1^ reported for *P. thermoglucosidasius* strain TM242 [9].

Recently, hot microbubble clouds generated by fluidic oscillation in shallow liquids (liquid height approximately a few bubble diameters) were shown to be effective in stripping ethanol from concentrated ethanol–water mixtures (90% (mol/mol) [17], 50% (mol/mol) [18] and 40% (wt/wt)^19^) due to high interfacial area for mass transfer, improved mixing in both phases and non-equilibrium mass transfer [17], [18], [19]. In our previous work [20], with a batch stripping system containing dilute ethanol–water mixtures (~4% (v/v)), it was demonstrated that short bubble residence times in the liquid phase are beneficial for ethanol separation, as non-equilibrium mass transfer leads to supersaturated vapour at the point at which the bubbles escape the liquid. It was also found that increasing the gas temperature (in the range of 90 to 150 °C) was beneficial for improving the ethanol removal rate depending on the liquid heights used (5 to 50 mm). Microbubble air stripping has been identified as a promising approach for removing volatile products from bioreactors with negligible effects on the liquid temperature [21] and low-shear mixing [12]. However, stripping ethanol from dilute ethanol–water mixtures with hot microbubbles where ethanol is continuously generated within the system or added externally has not been previously reported.

Therefore, the purpose of this study was to establish whether hot microbubble air stripping can be used to maintain ethanol concentrations below the toxicity threshold for TM242 and to find the maximum ethanol productivity of the microorganism that can be supported by this approach. A custom-made microbubble stripping unit (MSU) that can tightly control the gas and liquid temperatures has been used for this purpose. To mimic ethanol generation within a fermenter, ethanol was added to the MSU continuously at a rate that is similar to the ethanol generation expected from microbial strain TM242. A lump parameter model has been developed and validated to predict stripping performance of the system studied.

## 2 Materials and methods

### 2.1 Microbubble stripping unit

**Fig. 1:**
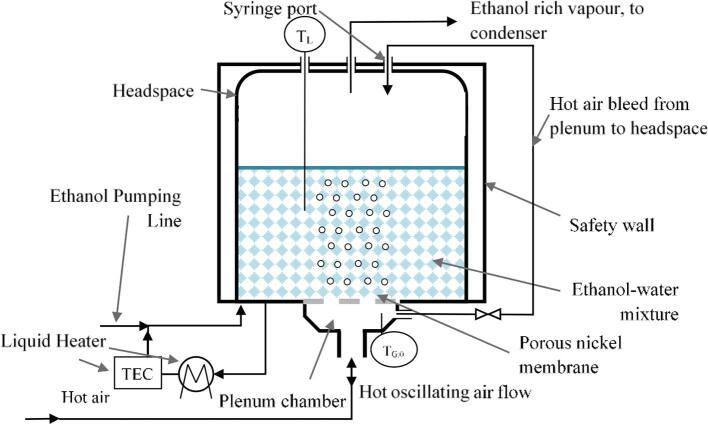
A schematic representation of the microbubble stripping unit (MSU) with continuous ethanol addition.

The design, build and operation of the microbubble stripping unit (MSU) is adapted from our previous work on batch stripping of dilute ethanol–water mixtures [20] and is summarised here. This unit was specifically designed to produce a dense cloud of hot microbubbles in a continuous liquid phase. A schematic diagram is shown in Fig. 1.

The MSU is well described as a cylindrical tank, which contains the ethanol–water mixtures surrounded by a square tank acting as a safety wall. The cylindrical section of the MSU is formed by a glass cylinder (borosilicate, Scott Glass Ltd.) which has a 140 mm internal diameter and a height of 120 mm. The glass cylinder was clamped between grooves in the stainless-steel support ring and the acetate lid which contained silicone seals to form an air-tight vessel. The microbubbles were produced within the cylindrical tank using a combination of a fluidic oscillator [22], [23] and a porous nickel porous membrane [24] (Micropore Ltd.) which had an average pore size of 20 µm and a 180 µm hexagonal pitch. The membranes were soldered onto copper supports to improve ability to seal the vessel. The plenum chamber for the gas inlet was designed to withstand gas temperatures up to 180 °C, and thus the base of the unit into which the stainless-steel support fitted was made using PTFE. As an additional safety feature, and in order to aid the optical measurements of the bubble size distributions, the external walls of the vessel were made into a square channel using glass. This allowed the capture of any liquid, should the glass cylinder crack, and allowed the glass cylinder to be removed for the bubble size measurements without optical aberrations. The acetate lid of the unit had ports for liquid sampling, temperature measurements and vapour removal.

The unit was designed to operate with liquid at 60 °C, in the optimum growth temperature range for *P. thermoglucosidasius*. The liquid temperature within the unit was maintained by circulating liquid through two heaters at a flow rate of 720 ml min^−1^. The second heating unit was operated with a PID controller using an RTD sensor (PT100) positioned inside the tank, approximately half-depth of the initial liquid height. To represent the ethanol production that would be expected from fermentation, an ethanol–water mixture containing 5% or 10% ethanol (v/v) was continuously added to the MSU with the recirculating liquid from the heating units.

### 2.2 Experimental setup

**Fig. 2:**
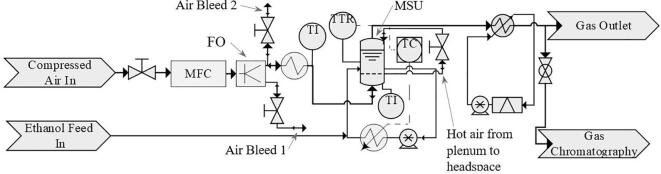
Process flow diagram (PFD) of the microbubble stripping process with continuous ethanol addition.

Prior to each experiment, membranes were cleaned with 2 M citric acid (99.8% monohydrate powder, Fisher Scientific) followed by 4 M sodium hydroxide (98.7%+ pellets, Fisher Scientific), both cleaning stages involved 15-minutes each. The experimental setup involving the assembled MSU and the auxiliary units is shown in Fig. 2. Compressed air was supplied to a fluidic oscillator (FO) at 150 SLPM (standard litres per minute) to produce an oscillating flow, controlled by a mass flow controller (Alicat, MCR series). The fluidic oscillator was connected with a feedback loop length of 2.5 m (i.d. = 5 mm), which sets the oscillation frequency at 73.5 Hz as measured using a pressure transducer (Hydrotechnik, HT-PT series) connected to a PC-oscilloscope (PicoScope, 6402C). The oscillation frequency was determined by analysing the pressure data using FFT power spectrum filtering, implemented in MATLAB (2015b).

The fluidic oscillator used in this study was a pilot-scale device which operated at flow rates well in excess of what was required. Therefore, only one outlet port (~75 SLPM) was used, and a large portion (~60 SLPM) was bled to the atmosphere. The rest of this air stream was passed through an air heater which consisted of an in-house built gas heating unit. The gas heater consisted of a stainless-steel pipe (L = 750 mm, o.d. = 6 mm, i.d. = 4 mm) coiled around a copper core housing a cartridge heater (120 V & 15 mm, RS-Pro). The cartridge heater was powered by an in-house built on/off control unit, which monitored the copper core temperature using a thermocouple (K-type) and maintained a constant temperature ± 3 °C. The coil temperature was adjusted to maintain a gas temperature in the plenum chamber at 120 °C for all experiments. The plenum gas temperature was measured using a thermocouple (K-type) placed close to the membrane without making contact and was logged manually once every minute (0.017 Hz).

As a safety feature, the air heater was positioned above the liquid level to reduce the probability of the liquid entering the heating unit. A gas flow rate of ~15 SLPM was passed through the heater to the MSU, then larger proportion of gas was directed from the plenum chamber to the headspace of the unit. This flow arrangement served two purposes. First, the line connecting the gas heater to the MSU was of a length of 0.95 m, which led to a high gas residence time and a noticeable heat loss at low gas flow rates of ~1 SLPM required for bubbling. Therefore, high gas flow rates to the plenum were beneficial in achieving the required gas temperature. Second, high vapour residence times in the headspace promoted significant condensation; therefore, heated air supplied to the headspace (~14 SLPM) via the plenum chamber reduced residence time of gas/vapour in the sparger and the headspace. Accurate gas flow rate measurements through the porous membrane (for bubble generation) were difficult due to this gas flow arrangement and the oscillatory nature of the flow. However, the gas flow rate used for microbubble production was kept constant at 1 VVM (volume of gas flow per volume of liquid per minute) for each experiment, at an inlet gas temperature of 120 °C, measured using the displacement method.

Prior to each experiment fresh 2% (v/v) ethanol–water mixtures were prepared by mixing pure ethanol (Fisher Scientific, 99.8%+) with deionised water. This mixture was heated to 60 °C and introduced to the MSU once the gas flow to the plenum chamber has reached the desired temperature (120 °C). This allowed immediate start of the experiment minimising evaporation before data collection and prevented liquid seepage to the plenum chamber. The vapour stream leaving the MSU was passed through two glass condensers in series (double-walled and internal coiled, Quick-Fit) which were dried with hot air before the start of the experiment and were cooled by a diluted (35% [v/v]) automobile coolant/antifreeze concentrate with added corrosion inhibitor (Halfords, UK). This liquid was maintained at −10 °C by a refrigeration unit (LABPLANT, PB-80/2 Refrigeration Bath, UK).

To simulate continuous ethanol production of a fermenting microorganism, an additional ethanol source was included. This was done by adding 5 or 10% (v/v) ethanol/water mixtures into the circulation loop at a dilution rate of 30% of the MSU volume per hour ($R_{\mathrm{feed}}$= 0.3 h^−1^) by a piston pump. In total, six experimental operating conditions were investigated. The initial liquid heights (*H_0_*) in the MSU were set to 10, 25 and 50 mm while the ethanol concentrations in the pumping line was set to 5 and 10% (v/v). This initial liquid height range were chosen based on our previous batch stripping experiments, and the value *H_0_* = 35 mm was estimated to be the liquid height that would produce no liquid volume change based on the average evaporation rate (approximately 170 ml/h) reported in the batch study [20]. All stripping experiments were repeated 6 times.

### 2.3 Experimental measurements

#### 2.3.1 Concentration measurements

Samples of the ethanol/water mixtures contained within the MSU and the condenser system were taken every 15 min for concentration measurements. When recovering condensate, the condensers were shaken to dislodge as much liquid as possible from the internal surfaces prior to sampling. The condensate was collected into a jar which was weighed to record the quantity of condensate recovered. To analyse the ethanol concentration, gas chromatography was used. 1 ml was transferred into a chromatography vial followed by a propanol/water mixture which constituted the internal standard. The propanol (99.9%+) was purchased from Fisher Scientific. For the liquid measurements, 100 µL of 40% (v/v) internal standard was used. For the condensate, 200 µL of 80% (v/v) internal standard was used. The difference in procedure was aimed at retaining appropriate precision for the expected higher ethanol concentration in the condensate. The liquid samples were analysed by gas chromatography (Agilent 7890A) with a J&W (DB-WAX) column (Agilent Technologies, 30 m × 0.250 mm with 0.25 μm coating) with an injection temperature of 150 °C and an oven temperature of 45 °C using a 1 ml/min helium mobile phase. Each sample was injected five times and the average ratio between the propanol and ethanol peak was used to calculate the concentrations by comparing with calibration tests done with ethanol/water standards (20, 10, 5 & 2.5% [v/v]). Despite using two condensers operated well below 0 °C, mass balances over the system revealed noticeable loss of ethanol vapour. Therefore, an additional figure is produced in the analysis whereby water is assumed to be fully recovered and the measured concentrations of the condensate were adjusted to satisfy the mass balance to estimate the ‘probable’ ethanol concentrations in the vapour phase [25].

#### 2.3.2 Bubble size measurements

The microbubbles produced in the MSU were recorded using a high-speed camera (Photron Fastcam, M2.1) with an Infinity KC long-distance lens. The light source used was an LED lamp (Kern, Dual Fiber Unit LED). In order to remove any optical distortion, the glass cylinder was removed, and the square outer channel was used to contain the liquid. The bubbles were recorded with 0 to 5% (v/v) ethanol–water mixtures. The videos were analysed using the software ImageJ to calculate the Feret Diameter (bubbles that were unclear, blurred or with circularity less than 0.7 were neglected) which provided bubble size distributions. The membrane was cleaned before each experiment as described earlier. The lens was focussed onto various positions in the viewing plane, just above the membrane (~3–4 mm), to give a reasonable representation of the bubbling from across the entire membrane. Approximately 425 photos containing over 10,000 bubbles were processed.

## 3 Results and discussion

**Fig. 3:**
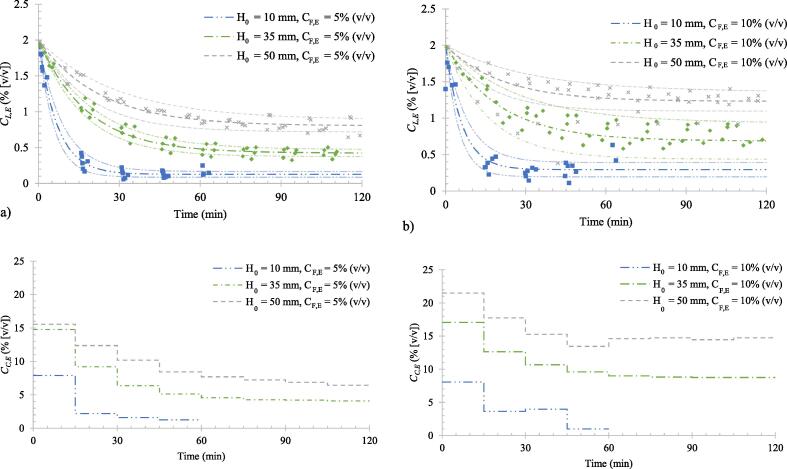
Concentration profiles for continuous hot microbubble air stripping. Liquid composition for adding ethanol–water mixtures with concentration a) 5% (v/v) and b) 10% (v/v). Adjusted condensate composition for c) 5% & d) 10% dilution. The faded lines indicate two standard errors of the experimental data (Eq. (7), presented later).

Fig. 3 shows the concentration profiles in the liquid contained in the microbubble stripping unit (MSU) and the condensate collected from the condenser system over time. In all cases the liquid composition decreased from the initial value of 2% (v/v) to a steady state value in times ranging from ~ 30 min (for initial liquid height, *H_0_* = 10 mm) to ~ 90 min (for *H_0_* = 50 mm). This indicates that ethanol is evaporated preferentially to water in every case. Regardless of the ethanol concentration added (5% or 10% (v/v)) to the MSU, higher liquid heights resulted in higher steady state ethanol concentrations and took longer to reach the steady state ethanol concentration. The initial amount of ethanol in the MSU and the ethanol addition rate is proportional to *H_0_*; therefore, reduction in the concentration is relatively slower for the higher liquid heights. However, high ethanol concentrations in the MSU are beneficial for achieving a higher stripping rate as the concentration driving force leads to high mass transfer rates. A steady state concentration is reached once the ethanol addition rate is equal to the ethanol removal rate by microbubble stripping. Therefore, the steady state concentration reached for high liquid heights (i.e. high addition rates) tended to be higher, to maintain a high stripping rate required to balance the high ethanol addition rates. This effect is evident in Fig. 3(a) and (b) where doubling the addition rate (from 5%(v/v) to 10%(v/v)) leads to elevated steady state concentrations for all liquid heights.

The ethanol concentrations in the vapour phase presented in Fig. 3(c) and (d) are average values of condensate collected over 15-minute time periods. These values were adjusted following a mass balance to account for the minor, but inevitable, ethanol loss from the condensers. These condensate concentrations follow the same trend observed with the liquid concentrations and the vapour concentration profiles appear in the same order (in terms of magnitude) as the liquid profiles. As the liquid concentration drops, vapour produced from it becomes less rich in ethanol; therefore, the faster drop in liquid concentrations observed with *H_0_* = 10 mm is associated with low vapour concentrations. In our previous work involving batch operation of the MSU [20], it was demonstrated that the liquid height affects both the stripping rate and the concentration of vapour produced from that liquid. If the liquid height in the MSU is selected such that the vapour has not reached thermal equilibrium with the liquid, it is possible to achieve slightly higher vapour concentrations than that predicted at equilibrium.

The concentration profiles with respect to time plotted by data fitting conform well with the exponential decay curves reaching steady state values, as shown by Fig. 3(a) and (b). These profiles contain two key pieces of information: first, the steady state concentration reached (*C_L,E,∞_*), and second, the time taken to reach an approximate steady state concentration. The bubble size distributions based on image analysis found that the average bubble size within the MSU was 222 µm across all concentrations with a coefficient of variation of 38%. This result allowed the assumption of uniform vapour concentration inside the bubbles leaving the process due to well mixed conditions [21]. The expected line of best fit can therefore be described by an ethanol molar balance over the system, assuming equilibrium,

(1) $$\frac{d\left(C_{L,E}V_{L}\right)}{\mathrm{dt}}=N_{+}-N_{-}$$

where *C_L,E_* represents the ethanol concentration in the liquid, *V_L_* represents the liquid volume and *N_+_* and *N_-_* represent the ethanol molar flows into and out of the liquid phase, respectively. If the only method by which ethanol can leave the system is by microbubble stripping, and the exit gas stream is equilibrated both in terms of the gas temperature and concentrations of water and ethanol, the vapour pressure of ethanol leaving the liquid phase ($P_{E}^{*}$) can be calculated using the modified Raoult’s Law.

(2) $$P_{E}^{*}=\gamma_{E}x_{E}P_{E}^{0}$$

where *γ_E_* is the activity coefficient of ethanol, which was calculated using the NRTL method [26], [27] and is considered a constant over the small ethanol concentration range studied. The saturated vapour pressure (*P_E_*^0^) was determined using Antoine equations [28] based on the interface temperature of the bubbles [20], [29]. The water concentration *C_L,W_* is much larger than the ethanol concentration in the dilute ethanol mixtures considered in this study; therefore, the mole fraction of ethanol $x_{E}$, can be simplified as: $x_{E}=C_{L,E}/\left(C_{L,E}+C_{L,W}\right)\approx C_{L,E}/C_{L,W}$. By considering the equilibrium gas phase ethanol concentration ($C_{E,G}^{*}$) and the gas flow rate (${\dot{V}}_{\mathrm{air}}$), the molar flow rate of ethanol leaving the liquid phase can be written as:

(3) $$N_{-}={\dot{V}}_{\mathrm{air}}C_{G,E}^{*}={\dot{V}}_{\mathrm{air}}\gamma_{E}\frac{C_{L,E}P_{E}^{0}}{C_{L,W}RT_{L}}$$

where $T_{L}$ is the liquid temperature and R is the universal gas constant. The concentration of water in eq. (3) can be expressed using density ($\rho_{W}$) and its relative molar mass ($M_{r,W}$). The inflow of ethanol to the MSU via the pumping line can be calculated from the volumetric flow rate (${\dot{V}}_{F}$) and the feed ethanol concentration ($C_{F,E}$). By substituting these quantities to (1):

(4) $$\frac{d\left(C_{L,E}V_{L}\right)}{\mathrm{dt}}={\dot{V}}_{F}C_{F,E}-{\dot{V}}_{\mathrm{air}}\gamma_{E}C_{L,E}\frac{M_{r,W}P_{E}^{0}}{\rho_{W}RT_{L}}=A-BC_{L,E}$$

where constants A and B can be defined as, $A={\dot{V}}_{F}C_{F,E}$ and $B={\dot{V}}_{\mathrm{air}}\gamma_{E}\frac{M_{r,W}P_{E}^{0}}{\rho_{W}RT_{L}}$. Now, depending on the level of change in *V_L_* during the experiment, two cases can be considered.

Case I - *V_L_* remains nearly constant during the experiments: For instance, $H_{0}=35$ mm where liquid addition rate is approximately equal to the liquid evaporation from the system. By integration of eq. (4) from the start of the stripping process to anytime *t,* with initial condition $C_{L,E}\left(0\right)=C_{L,E,0}$:

(5) $$C_{L,E}=\frac{1}{B}\left[A-\left(A-BC_{L,E,0}\right)e^{-\frac{B}{V_{L}}t}\right]$$

At steady state, *C_L,E_*(*t*→∞) = *C_L,E,∞_*. From eq. (5)

$C_{L,E,\infty }=\frac{A}{B}$*;* then *(*6*)*

(7) $$C_{L,E}=C_{L,E,\infty }-\left(C_{L,E,\infty }-C_{L,E,0}\right)e^{-kt},$$

where, $k=\frac{\gamma_{E}{{\dot{V}}_{\mathrm{air}}M}_{r,W}P_{E}^{0}}{V_{L}\rho_{W}RT_{L}}$.

Case II - *V_L_* is changing with time but the overall evaporation rate remains constant (i.e. $dV_{L}/dt=\alpha$), then eq. (4) becomes

(8) $$V_{L}\frac{dC_{L,E}}{\mathrm{dt}}+C_{L,E}\alpha =A-BC_{L,E}$$

Substituting $V_{L}=V_{L,0}+t\alpha$ and rearranging, eq. (8) becomes

(9) $$\frac{1}{A-\left(B+\alpha \right)C_{L,E}}\frac{dC_{L,E}}{\mathrm{dt}}=\frac{1}{\left(V_{L,0}+t\alpha \right)}$$

Integrating eq. (9), given $C_{L,E}\left(t=0\right)=C_{L,E,0}$ gives

(10) $$C_{L,E}=\frac{A}{B+\alpha }-{\left(\frac{A}{B+\alpha }-C_{L,E,0}\right)\left(1+\frac{\alpha }{V_{L,0}}t\right)}^{\frac{-(B+\alpha )}{\alpha }}$$

The curves of best fit shown in Fig. 3(a) and (b) are those predicted by eq. (7), with fitted values for the steady state concentration and the decay constant. Generally, these curves represent the experimental data well, except for the final data point for $H_{0}$ = 10 mm for both $C_{F,E}$ = 5 and 10% (v/v). For this lowest liquid height tested, reduction in liquid height with time leads to uneven distribution of liquid over the bubbling membrane after ~ 1 h and the liquid remaining in the unit is insufficient to cover the entire membrane uniformly, causing under-saturated vapour. This leads to inefficient stripping, and the continuous addition of ethanol to the unit via the pumping line causes $C_{L,E}$ to rise at the end. At this point, the experiments were terminated for $H_{0}$ = 10 mm. The volume percentage change per hour due the imbalance in evaporation and liquid addition to the unit for experiments run with $H_{0}$ = 10, 35 and 50 mm are estimated to be −61%, 0% and 9% respectively. These values correspond to liquid height changes of −9.5, 0, 2.5 mm per hour in the unit, respectively.

**Fig. 4:**
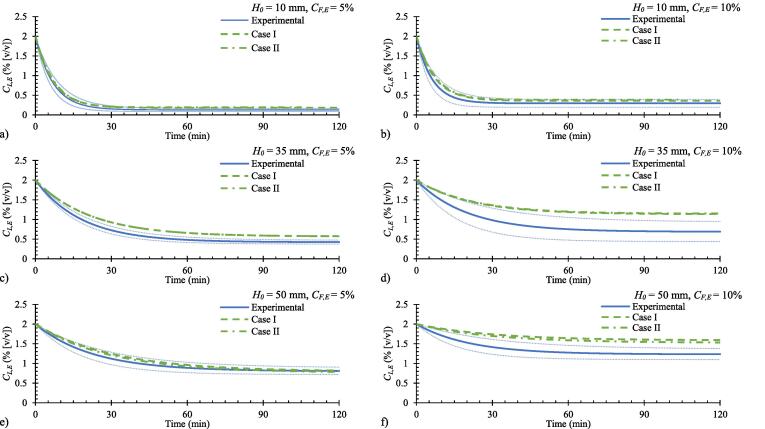
Comparison of the liquid ethanol concentration predicted by eq. (7) (Case I) and eq. (10) (Case II) and the average curves fitted to the experimental data. The faded lines represent two standard errors of the experimental data.

Fig. 4 shows a comparison of curves predicted for case I and case II (i.e. based on eqs. (7), (10)) along with the fitted profiles for experimental data of the liquid ethanol concentration over time. In all cases the difference between the two models was found to be negligible; therefore, only eq. (7) was used for data fitting in Fig. 3.

Overall, the model predictions and the experimental curves agree well, both the profile features and the magnitudes, which demonstrates the suitability of the model for predicting the dynamic behaviour of the system. However, there are a few exceptions. For instance, measured liquid ethanol concentration profiles for $H_{0}$ = 35, 50 mm and $C_{F,E}$ = 10% (v/v) is lower than that predicted by eq. (7). This could be due to a combination of several factors. As mentioned previously, there is a possibility of achieving higher than equilibrium vapour concentrations, if the liquid height remains sufficiently low to avoid thermal equilibrium. With computational modelling, Zimmerman et al. demonstrated that the average bubble temperature and the average water concentration changes significantly within the first few milliseconds of contacting with a liquid [21]. In our previous study, we demonstrated the significance of interface temperature on heat and mass transfer through gas–liquid interfaces [20]. As the interface temperature is likely to vary during this initial period [29], [30], because both evaporation and sensible heat transfer take place simultaneously, key parameters in eq. (4) will change: namely the activity coefficient (γ) and the saturated vapour pressure ($P_{E}^{0}$). The interface temperature is therefore important in calculating the driving force for mass transfer, and thus the actual mass transfer rate could be different to the values calculated here using the bulk liquid temperature ($T_{L}$) [20]. The increased stripping rate associated with these effects would produce steady state concentrations less than values predicted using the modelling equations that uses equilibrium. Another factor for any discrepancy between the model predictions and the experimental curves may arise due to the practical difficulty of measuring the gas flow rate accurately in a ‘live’ experiment. In this experimental setup, gas flow to the MSU is oscillated to aid microbubble production; therefore, flow measurements are only estimates. From equation (6), $C_{L,E,\infty }=\frac{A}{B}=\frac{{\dot{V}}_{F}C_{F,E}\rho_{W}RT_{L}}{{\dot{V}}_{\mathrm{air}}\gamma_{E}M_{r,W}P_{E}^{0}}$. Assuming constant $\gamma_{E}$ for narrow concentration range considered and constant $T_{L}$, steady state concentration can be approximated as

(11) $$C_{L,E,\infty }=\frac{\rho_{W}RT_{L}}{\gamma_{E}M_{r,W}P_{E}^{0}}∙\frac{{\dot{V}}_{F}C_{F,E}}{{\dot{V}}_{\mathrm{air}}}\approx \chi \frac{{\dot{V}}_{F}C_{F,E}}{{\dot{V}}_{\mathrm{air}}}$$

where *χ* is a constant that describes the conditions and parameters of the system inside the MSU, particularly the liquid temperature. eq. (11) demonstrates that the steady state concentration is sensitive to the gas flow rate. It was estimated that ~20% variation of the gas flow rate would account for the maximum difference observed between the modelled and the experimental steady state ethanol concentrations.

Table 1 summarises the performance of the unit at different operating conditions, i.e. the quasi steady state ethanol concentration in the liquid phase and the ethanol removal rate from the system at different liquid heights. Note that the ethanol removal rate is equal to the ethanol addition rate as the system is at quasi steady state after 30–90 min of operation. Since the unit is operated with different liquid volumes, ethanol removal rate per unit volume is also included in the table. The ethanol addition rate to the unit for each case has been set proportionate to the initial liquid volume in the unit (dilution rate, $R_{\mathrm{feed}}=$0.3 h^−1^); therefore, higher ethanol removal rates (g h^−1^) can be achieved with larger liquid volumes in the MSU. However, the disadvantage in setting a higher removal rate is that the steady state ethanol concentration in the unit will be higher to compensate for increase in mass transfer rate required. It is also worth noting that the removal rate per unit volume (g L^−^^1^h^−1^) does not change noticeably with the liquid height and nearly doubled with doubling the feed concentration. The main motivation of this study was to find out whether hot microbubble stripping can be used to mitigate product inhibition in fermenters; therefore, results presented in Table 1 will be analysed with the context of microbial ethanol production.

**Table 1:** Ethanol removal rates and the steady state ethanol concentrations at various operating conditions.

$C_{F,E}$(%[v/v])	$H_{0}$(mm)	$V_{L,0}$(cm^3^)	Addition rate to MSU [$R_{\mathrm{feed}}$(h^−1^) × $V_{L,0}$(L) × $C_{F,E}$ (%[v/v]) × ρ_E_ (g L^−^^1^)] (g h^−1^)	Removal rate per unit volume (g L^−^^1^h^−1^)	$C_{L,E,\infty }$(%[v/v])
5	10	186	2.10	11.3	0.13
	35	571	6.80	11.9	0.42
	50	802	8.62	10.7	0.80
10	10	186	4.28	23.0	0.29
	35	571	14.9	26.0	0.69
	50	802	21.2	26.5	1.23

Product inhibition is a common issue in many batch fermentation processes. If the products that cause this inhibition could be removed continuously from the fermentation system, high productivities are possible. Cripps et al. [9] demonstrated that *P. thermoglucosidasius* (TM242) is capable of consuming a wide range of sugars found available in lignocellulosic feedstocks and reported ethanol productivities up to 3.2 g L^−^^1^h^−1^. However, it was anticipated that commercial fermentation systems would allow ethanol generation rates as high as 6–8 g L^−^^1^h^−1^, if produced ethanol can be removed from the fermenter continuously. This strain, TM242, starts to show a reduction in ethanol production rates at ethanol concentrations above ~2% (v/v) and cease production completely around 3.5–4% (v/v) [31]. Therefore, we were interested to find out whether hot microbubble stripping is capable of maintaining maximum ethanol concentration in the fermentation broth ~2% (v/v) by removing ethanol at the high rates of productivity mentioned above. We will consider two configurations: *in situ* stripping within a bioreactor and operating a bioreactor with a side-arm extractor (MSU). This analysis is limited to technical possibilities in terms of mass transfer and any other complications (eg foaming) that may arise in an actual fermentation system are not considered.

**Fig. 5:**
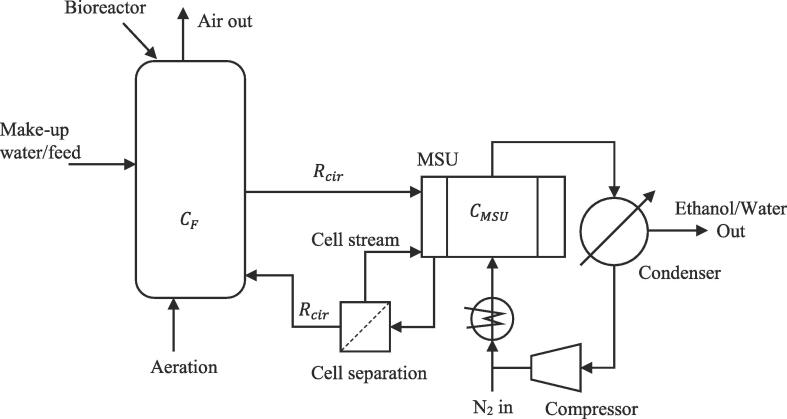
Suggested configuration for a side arm microbubble stripping process.

First, consider the MSU as a bioreactor with *in-situ* stripping. According to Table 1 all operating conditions could maintain the ethanol concentration well below the inhibitory level expected for TM242 while removing ethanol at high rates expected from improved thermophilic strains. The volumetric removal rates are insensitive to $H_{0}$ but depend on $C_{F,E}$. For instance, hot microbubbles can remove ethanol at a rate of ~11.3 g L^−^^1^h^−1^ for $C_{F,E}$ = 5% (v/v) and ~25.2 g L^−^^1^h^−1^ for $C_{F,E}$ = 10% (v/v), regardless of the liquid volume in the tank. These removal rates are much higher than the reported ethanol productivities for TM242. If the experiment adequately represents a fermentation system, no product inhibition would be expected, and thus the ethanol production rate should continue at the maximum rate if the ethanol addition rates are truly representative of the metabolic rate of the microorganism. However, integrating a stripping system inside a bioreactor could be difficult and could lead to various practical issues. For instance, bioreactors are equipped with agitators, baffles, spargers and various measurement probes; therefore, uninterrupted bubbling of a large cross-sectional area would be difficult. Also, high liquid heights in bioreactors could adversely affect the stripping rate. Most importantly, hot bubbles could adversely affect the physiology of the micro-organism, in which case product removal should be carried out in a separate unit with cell separation before feeding to MSU. Therefore, the most favourable configuration to operate a fermenter with continuous product removal is to couple it with an MSU as shown in Fig. 5.

This configuration is flexible enough to be operated with existing bioreactors and would allow operation of the MSU under the optimum conditions found in this study. Consider a 1 L bioreactor where the ethanol concentration is to be maintained at 2% (v/v), or $C_{F}$ = 15.2 g L^−1^, where ethanol inhibition is insignificant. Maintaining the highest possible ethanol concentration in the bioreactor, without product inhibition, is advantageous for high quality stripping as there should be a sufficient concentration difference between the units. If the concentration difference between the MSU and the bioreactor is relatively small, the liquid recirculation rate ($R_{\mathrm{cir}}$) required would be exceptionally high, increasing the operating costs. Furthermore, the residence time of the broth in the MSU could also be too low for stripping to complete. In selecting a liquid height to operate the MSU, both the steady state concentration and the removal rate (g h^−1^) should be considered simultaneously. Since we are interested in finding out the maximum potential of the unit, consider the high ethanol addition (generation) case, i.e. $C_{F,E}$ = 10% (v/v) where up to 21.2 g h^−1^ is possible. Even though $H_{0}$ = 50 mm offers the highest ethanol removal rate (21.2 g h^−1^), the steady state concentration under these conditions (1.23% (v/v)) would be too close to the concentration in the fermenter. Therefore, $H_{0}$ = 35 mm that maintains an ethanol concentration of 0.69% (v/v), or $C_{\mathrm{MSU}}$ = 5.2 g L^−1^, and removes ethanol at a rate of 14.9 g h^−1^ was selected. According to the concentration profile for $H_{0}$ = 35 mm shown in Fig. 3(b), the residence time required to reduce the concentration from 2% (v/v) to 0.69% (v/v) would be approximately 45 min. Therefore, the liquid recirculation rate ($R_{\mathrm{cir}}$) is limited to a maximum of ~ 13 ml min^−1^. The maximum productivity supported by the unit can now be found by an ethanol balance over the fermenter (eq. (12)).

(12) $$V_{F}{\hat{P}}_{E}=R_{\mathrm{cir}}\left(C_{F}-C_{\mathrm{MSU}}\right)$$

where $V_{F}$ is the fermenter liquid volume (L), ${\hat{P}}_{E}$ is the maximum productivity of the microorganism (g L^−1^ h^−1^) and $C_{F}$ and $C_{\mathrm{MSU}}$ are steady state concentrations (g L^−1^) in the fermenter and MSU, respectively. According to Equation (12), the maximum ethanol productivity would be 7.8 g L^−^^1^h^−1^. If the maximum ethanol productivity of the microorganism is less than 7.8 g h^−1^, higher fermentation broth volumes (>1 L) can be processed or $R_{\mathrm{cir}}$ can be reduced. However, if the productivity of the organism is higher than 7.8 g L^−^^1^h^−1^, broth volume of the fermenter should be reduced or two (or more) MSU can be linked to the fermenter. For instance, if the MSU is operated with a 0.5 L fermenter, ${\hat{P}}_{E}$ of ~14.9 g L^−^^1^h^−1^ can be processed by the unit.

Therefore, operating parameters can be chosen based on eq. (12) to produce the same quality of separation depending on the application. It is also worth noting that the maximum fermenter volume that can be coupled with the MSU depends on the ethanol productivity within the bioreactor and can be found by the equation VFP^E = 14.9, where $V_{F}$ and ${\hat{P}}_{E}$ are specified in L and g L^−^^1^h^−1^ respectively. If the ethanol productivity is less than this value, larger broth volumes can be processed by the unit. In designing an MSU for dilute ethanol mixtures, the liquid height in the unit should be kept in line with the optimum heights found in this study. Therefore, it is anticipated that scaling up stripping for larger fermenters can be done by either increasing the cross-sectional area for bubbling or adding more stripping units to the process. Even though the results found in this study looks promising, experiments and mathematical modelling were related to pure ethanol–water mixtures mimicking a fermentation system. Further work with an actual fermentation system is needed to identify practical issues and mass transfer performance in fermentation broths.

## 4 Conclusions

A hot microbubble stripping unit (MSU) designed for product removal from bioethanol fermentation has been studied with dilute ethanol–water mixtures at 2% (v/v) maintained at 60 °C. Ethanol was added to the system at a rate of 2.1–21.2 g h^−1^ to imitate the ethanol production of the microorganism *P. thermoglucosidasius* (TM242). Both the initial liquid height in the unit and the ethanol addition rate affected the steady state ethanol concentration reached, but in all cases ethanol concentration was maintained well below the threshold for TM242 to produce optimally, which is ~2% (v/v). High ethanol addition/generation rates are advantageous for achieving high stripping rates from the unit as the driving force for mass transfer increases with the concentration, but the steady state ethanol concentration rises with addition/generation rate. The ethanol concentration profiles within the stripping unit were useful in determining the residence time of the liquid for achieving steady state operation. A 0.5 L fermenter that operates in combination with this microbubble stripping unit can support ethanol productivities up to 14.9 g L^−^^1^h^−1^ for TM242, which is commercially competitive. Productivities lower than this maximum value can be processed by either increasing the fermenter liquid volume or by adjusting the recirculation rate between the units. This study demonstrates technical feasibility of continuous product removal from a simulated fermenter, but further work is necessary with an actual fermentation system to determine the applicability of this novel approach.

## Declaration of Competing Interest

The authors declare that they have no known competing financial interests or personal relationships that could have appeared to influence the work reported in this paper.

## References

[1] Sonego J.L.S., Lemos D.A., Pinto C.E.M., Cruz A.J.G., Badino A.C. (2016). Extractive fed-batch ethanol fermentation with CO2 stripping in a bubble column bioreactor: Experiment and modeling. Energy Fuels.

[2] Amorim H.V., Lopes M.L., de Castro Oliveira J.V., Buckeridge M.S., Goldman G.H. (2011). Scientific challenges of bioethanol production in brazil. Appl. Microbiol. Biotechnol..

[3] Ciesielski A., Grzywacz R. (2019). Dynamic bifurcations in continuous process of bioethanol production under aerobic conditions using saccharomyces cerevisiae. Biochem. Eng. J..

[4] Azhar S.H.M., Abdulla R., Jambo S.A., Marbawi H., Gansau J.A., Mohd Faik A.A., Rodrigues K.F. (2017). Yeasts in sustainable bioethanol production: A review. Biochem. Biophys. Reports.

[5] Baeyens, J.; Kang, Q.; Appels, L.; Dewil, R.; Lv, Y.; Tan, T. Challenges and Opportunities in Improving the Production of Bio-Ethanol. Progress in Energy and Combustion Science. Pergamon April 2015, pp 60–88. https://doi.org/10.1016/j.pecs.2014.10.003.

[6] Onuki, S.; Koziel, J. A.; Jenks, W. S.; Cai, L.; Grewell, D.; van Leeuwen, J. H. Taking Ethanol Quality beyond Fuel Grade: A Review. Journal of the Institute of Brewing. John Wiley and Sons Inc. October 2016, pp 588–598. https://doi.org/10.1002/jib.364.

[7] Carroll A., Somerville C. (2009). Cellulosic biofuels. Annu. Rev. Plant Biol..

[8] Shrestha D.S., Staab B.D., Duffield J.A. (2019). Biofuel impact on food prices index and land use change. Biomass Bioenergy.

[9] Cripps R.E., Eley K., Leak D.J., Rudd B., Taylor M., Todd M., Boakes S., Martin S., Atkinson T. (2009). Metabolic engineering of geobacillus thermoglucosidasius for high yield ethanol production. Metab. Eng..

[10] Sun Y., Cheng J. (2002). Hydrolysis of lignocellulosic materials for ethanol production: A review. Bioresour. Technol..

[11] Kumar S., Dheeran P., Singh S.P., Mishra I.M., Adhikari D.K. (2015). Continuous ethanol production from sugarcane bagasse hydrolysate at high temperature with cell recycle and in-situ recovery of ethanol. Chem. Eng. Sci..

[12] Ribeiro, C. P.; Lage, P. L. C. Gas-Liquid Direct-Contact Evaporation: A Review. Chem. Eng. Technol. 2005, 28 (10), 1081–1107. https://doi.org/10.1002/ceat.200500169.

[13] Durkee, E. L.; Lowe, E.; Baker, K. A.; Burgess, J. W. Field Tests of Salt Recovery System for Spent Pickle Brine. J. Food Sci. 1973, 38 (3), 507–511. https://doi.org/10.1111/j.1365-2621.1973.tb01468.x.

[14] Fleming H.P., Daeschel M.A., McFeeters R.F., Pierson M.D. (1989). Butyric acid spoilage of fermented cucumbers. J. Food Sci..

[15] Zaida A.H., Sarma S.C., Grover P.D., Heldman D.R. (1986). Milk concentration by direct contact heat exchange. J. Food Process Eng..

[16] Taylor F., Marquez M.A., Johnston D.B., Goldberg N.M., Hicks K.B. (2010). Continuous high-solids corn liquefaction and fermentation with stripping of ethanol. Bioresour. Technol..

[17] Abdulrazzaq N.N., Al-sabbagh B.H., Rees J.M., Zimmerman W.B.J. (2016). Separation of azeotropic mixtures using air microbubbles generated by fluidic oscillation. Sep. Mater. Devices Process..

[18] Abdulrazzaq N.N., Al-Sabbagh B.H., Rees J.M., Zimmerman W.B. (2016). Purification of bioethanol using microbubbles generated by fluidic oscillation: A dynamical evaporation model. Ind. Eng. Chem. Res..

[19] Al-yaqoobi A., Hogg D., Zimmerman W.B. (2016). Microbubble distillation for ethanol-water separation. Int. J. Chem. Eng..

[20] Calverley J., Zimmerman W.B., Leak D.J., Bandulasena H.C.H. (2020). Hot microbubble air stripping of dilute ethanol-water mixtures. Ind. Eng. Chem. Res..

[21] Zimmerman W.B., Al-Mashhadani M.K.H., Bandulasena H.C.H. (2013). Evaporation dynamics of microbubbles. Chem. Eng. Sci..

[22] Zimmerman W.B.J., Tesař V., Butler S., Bandulasena H.C.H. (2008). Microbubble generation. Recent Patents Eng..

[23] Zimmerman W.B., Hewakandamby B.N., Tesař V., Bandulasena H.C.H., Omotowa O.A. (2009). On the Design and Simulation of an Airlift Loop Bioreactor with Microbubble Generation by Fluidic Oscillation. Food Bioprod. Process..

[24] Wright A., Bandulasena H., Ibenegbu C., Leak D., Holmes T., Zimmerman W., Shaw A., Iza F. (2018). Dielectric barrier discharge plasma microbubble reactor for pretreatment of lignocellulosic biomass. AIChE J..

[25] Schläfle S., Senn T., Gschwind P., Kohlus R. (2017). Feasibility and energetic evaluation of air stripping for bioethanol production. Bioresour. Technol..

[26] Hadrich B., Kechaou N. (2010). Identification of best model for equilibrium data of ethanol - water mixture. J. Chem. Chem. Eng..

[27] Gmehling, J. A.; Onken, U. A.; Arlt, W. Vapor-Liquid Equilibrium Data Collection; Chemistry data series; Deutsche Gesellchaft fur Chemisches Apparatewesen: Frankfurt, 1997.

[28] Perry R.H., Green D. (1997). Perry’s Chemical Engineers’ Handbook.

[29] Guy C., Carreau P.J., Paris J. (1992). Heat and mass transfer between bubbles and a liquid. Can. J. Chem. Eng..

[30] Treybal, R. E. Mass Transfer Operations, 3rd ed.; Brown, J. V, Eichberg, M., Hess, C., Eds.; McGraw-Hill Inc., 1980.

[31] Ortenzi M. (2020).

